# The Proper Diet and Regular Physical Activity Slow Down the Development of Parkinson Disease

**DOI:** 10.14336/AD.2021.0123

**Published:** 2021-10-01

**Authors:** Patryk Andrzej Chromiec, Zofia Kinga Urbaś, Martyna Jacko, Jan Jacek Kaczor

**Affiliations:** ^1^Aronpharma Sp.z.o.o, Trzy Lipy 3, 80-172, Gdansk, Poland.; ^2^Department of Bioenergetics and Physiology of Exercise, Medical University of Gdansk, Debinki 7, Gdansk, 80-211, Poland.

**Keywords:** Parkinson’s disease, physical exercise, diet, neuroinflammation, neuroprotection

## Abstract

From year to year, we know more about neurodegeneration and Parkinson’s disease (PD). A positive influence of various types of physical activity is more often described in the context of neuroprotection and prevention as well as the form of rehabilitation in Parkinson’s patients. Moreover, when we look at supplementation, clinical nutrition and dietetics, we will see that balancing consumed products and supplementing the vitamins or minerals is necessary. Considering the biochemical pathways in skeletal muscle, we may see that many researchers desire to identify molecular mediators that have an impact through exercise and balanced diet on human health or development of the neurodegenerative disease. Therefore, it is mandatory to study the potential mechanism(s) related to diet and factors resulted from physical activity as molecular mediators, which play a therapeutic role in PD. This review summarizes the available literature on mechanisms and specific pathways involved in diet-exercise relationship and discusses how therapy, including appropriate exercises and diet that influence molecular mediators, may significantly slow down the progress of neurodegenerative processes. We suggest that a proper diet combined with physical activity will be a good solution for psycho-muscle BALANCE not only in PD but also in other neurodegenerative diseases.

Neurodegenerative diseases (ND) are one of the main problems of an aging society that relies on neurodegeneration, which leads to neuron death. Parkinson’s disease (PD) is the second most common neurodegenerative disorder after Alzheimer’s disease (AD). The economic cost of PD is estimated to exceed $23 billion annually in the United States alone [1]. PD is a complex and highly prevalent neurodegenerative disorder characterized by disabling motor abnormalities such as bradykinesia, postural instability, and gait disorders, often leading to falls [2, 3]. There is also an increased risk of fractures, aggravated disability, poor quality of life, and reduced survival [4]. PD is characterized by the loss of dopamine (DA) due to the degeneration of substantia nigra pars compacta (SNpc) in dopaminergic neurons [5]. Conversely, in PD the early and preferential loss of DA in the dorsal basal ganglia leads to diminished automatic and increased cognitive (frontal cortex) control of motor movements. Consequently, individuals with PD must handle and sustain a more extensive cognitive load to execute either motor or cognitive tasks [6, 7].

It has been found that in PD exercise stimulates DA synthesis in remaining dopaminergic cells and thus reduces symptoms [8, 9]. Moreover, it has been suggested that five fundamental principles of exercise enhance neuroplasticity in Parkinson patients. They are as following: (a) intensive activity maximizes synaptic plasticity; (b) activity promotes greater structural adaptation; (c) activity that is rewarding increase DA levels and promotes learning/re-learning; (d) dopaminergic neurons are highly responsive to exercise and inactivity (“use it or lose it”), and (e) where exercise is introduced at an early stage of the disease, progression may be slower [9-11]. Furthermore, exercise may exert its neuroprotective action by attenuating oxidative stress, a mechanism that has frequently been considered in PD genesis. Besides, regular exercise may activate antioxidant enzymes including superoxide dismutase (SOD), glutathione peroxidase (GPx), and catalase (CAT) [10] in the CNS, reduce glutamate receptors involved in the excitotoxicity, and downregulate expression of genes involved in apoptosis such as Bcl-x and DP-5. It may also modify the relationship of the DAT transsynaptic against vesicular (DAT/vesicular monoamine transporter), which reduces the susceptibility of dopaminergic neurons to neurotoxins and cytosolic oxidation of DA [11].

During aging, mitochondria become more sensitive to the damages caused by oxidative stress because antioxidant systems are less effective. If mitochondria are not working in the physiological condition, it may lead to lipids and proteins peroxidation [12]. PD patients have more problems with impaired mitochondria than age-matched controls [13, 14]. The loss of mitochondria is associated with DA neurons’ loss, which is the major problem in PD. The disruption of mitochondria function is mostly measured by the activity of the electron transport chain (ETC) complexes [12]. The ETC, especially complex I in PD is inhibited by the same toxins, which induce motor dysfunctions and impair dopaminergic neurons. That fact may connect the mitochondrial dysfunction with PD and it is a good direction to investigate if we may stop or slow down this impairment by exercise [12].

Neurodegenerative impairment may connect with a cytokine-induced imbalance in the kynurenine (KYN) pathway. As this pathway provides the primary route for tryptophan (TRP) degradation, it plays a major role in the maintenance of serotonin (5-HT) synthesis and the critical balance between neurotoxic and neuroprotective metabolites. Chronic inflammation is commonly reported in severe cognitive deficits and may contribute to the pathogenesis of each of these disturbances. Consequently, the KYN pathway has become a prospective target for treatment interventions [15]. It is believed that the ketogenic diet, which seems to reduce glucose and calorie intake, may also affect the KYN pathway and brain excitability. It is also known that calorie restriction may have a neuroprotective/antiepileptic effect. KYN, synthesized from TRP, may be transformed into various metabolites. Some of them are considered neuroprotective (e.g., kynurenine acid (KYNA)), while others are neurotoxic (e.g., 3-hydroxykynurenine (3-HK) and quinolinic acid (QUIN)). Even though progress has been made in explaining the interaction of metabolites of the KYN pathway in health and disease, questions need to be answered [15].

Alternative strategies with proven anti-inflammatory benefits, such as regular exercise, may provide a simple and effective long-term strategy in treating and preventing associated disorders by influencing enzyme function and helping in the reestablishment of critical protein balances. In recent years, preclinical and clinical studies have highlighted the fundamental influence that the intestinal microbiota exerts on the gut-brain axis, which is now renamed “microbiota-gut-brain axis” [16]. Several microbially-derived molecules may control different gut functions, including metabolic, nutritional, and immune responses, and brain activity, giving rise to a microbiota-mediated bottom-up control of the CNS [17]. The gut microbiota may also indirectly influence glutamatergic pathways along the microbiota-gut-brain axis by controlling TRP metabolism. In the gut TRP, the essential amino acid, contributes to the synthesis of numerous bioactive molecules, including 5-HT, KYN, and indole derivatives, under direct and indirect microbiota control [18]. Microbiota may interact with the nervous system; perhaps the most obvious scenario would be through modulation of host neurotransmitters and/or related pathways. Bacteria have been found to have the capability to produce a range of major neurotransmitters like histamine, DA, gasotransmitters, neuropeptides, steroids, endocannabinoids and others [19].

## The beneficial effect of selected physical activity in PD

This paragraph will provide that regular physical activity is one of the most important aspects of healthy aging. Moreover, it was documented that physical activity may prevent and slow down many chronic diseases such as diabetes, strokes, osteoporosis, circulatory and nervous diseases, including PD [20-22]. Pharmacological therapies are primarily used for symptomatic control and provide only short-term benefits before the disease reaches a severe stage. Physical activity and exercise may provide low-cost and universally available aids for current PD therapies. Therefore, studying the effects of physical activity and exercise on PD is substantial [23]. Several large controlled clinical studies have shown that bradykinesia, balance, and muscle strength may be improved by continuous exercise in PD’s early stage [24]. In scientific reports, many forms of physical activity that may positively affect patients with PD are described.

### Aerobic exercise

One of the most effective aerobic exercise forms is training on a mobile treadmill, where the pace of walking or running and the slope of the surface can be adjusted. It has been shown that aerobic training ameliorates walking in PD patients and improves gait speed, step length, and extends the distance covered [25]. It may also have a beneficial effect on reducing the number of falls and improving a sense of balance. The other data have been reported that aerobic workout strengthens muscles and improves cardiorespiratory fitness [21, 26, 27].

### Nordic walking

Nordic walking, a new form of recreational sport, is recently gaining popularity. Although it was established in the 1920s in Finland, fashion has only come to it now. During nordic walking, the whole body is stimulated and engaged in the effort, and the upper limbs are strengthened more than when walking or running without sticks. Therefore, nordic walking is also used to treat patients with PD. It was observed that during the 6-week training, the walking speed has improved, and the quality of life in patients and their families has also effectively augmented [28-33].

### Resistance or strength training

Resistance or strength training aim is to increase muscle mass and strength. It is crucial for people who are ill, older or even suffering from PD because due to stiffness and slowness, they are exposed to significant muscle strength deficits. It turns out that the rehabilitation, including resistance training, meaningfully improves muscle strength, mobility, quality of life, and increases lean body mass in PD patients [34-37].

### Choreotherapy

Choreotherapy belongs to art therapy. It includes music, expressive movement, and dance. It is a psychotherapy method based on the idea of ??the indissolubility of the soul and body. It was noted that dance has a beneficial effect on mobility, walking speed, and PD patients’ balance. Moreover, it was found that choreotherapy can be an exciting substitute for traditional exercises used so far during rehabilitation [38]. Music, which is an external stimulus, facilitates the beginning of the movement, and the dance itself teaches a precise movement strategy. Dance may also deal with cognitive and psychological symptoms [39]. It has been shown that dance patterns involve motor planning and memory [39] and give an opportunity to practice multitasking, e.g., the spatial and temporal execution of steps in time to the music while ensuring postural stability [40-44].

### Tai Chi

Tai Chi is not only a system of stretching, balance, and coordination exercises combined with meditation, but also the art of self-defense. It was developed in the 11th century in China and is based on making slow, harmonious movements that have a positive impact on maintaining good health and well-being, and at the same time achieving control over your own body and mind. Regular exercises have a beneficial effect on stress reduction. Improvements in balance and mobility were noted in PD patients participating in a 12-week study, including 45 minutes session once a week [45]. However, in another study, there was no improvement in the Tai Chi group [46]. Any form of effort adjusted to the disease’s stage, physical and mental conditions of a PD patient, can be a source of benefits, not only physical but also psychological. Each physical activity contributes to improving our well-being, which also translates into our health [46-48]. Li and coworkers in cohort analysis showed that the incorporation of Tai Chi in PD patients’ daily lives allowed them to stay functionally and physically active. Moreover, it was found the improvement of physical parameters indicated, that Tai Chi have the potential to slow down the progression of PD and delay the administration of levodopa (L-DOPA) [49].

### Neuroprotective mechanisms induced by physical exercise

In a narrative review, Paillard et al. [50] summed up the neuroprotective mechanism induced by physical exercise (PE) in subjects affected by PD. The current knowledge about the mechanisms involved in the protective effect of PE against PD relies on data obtained in animal models. It has been shown that PE has a protective effect on the dopaminergic function in PD models by stimulating the expression of several neurotrophic factors and angiogenesis [51]. In response to PE, the concentration of DA increases, and this neurotransmitter’s receptors enhance their sensitivity [52]. More precisely, PE reduces the alteration of the dopaminergic neurons in the substantia nigra and contributes toward reconstituting the function of the basal ganglia involved in the motor command by the adaptive mechanisms of DA and glutamate neurotransmission [52]. This action is related to an increased concentration of brain-derived neurotrophic factor (BDNF) [53]. In addition, the hyperexcitability often observed in the basal ganglia is decreased [54].

Diminished loss of the neurons that produce DA is found in PD mice after 18 months of training, as well as there is observed an improvement of movement-balance coordination [55]. Mechanistic investigations revealed that the neuronal and behavioral recovery generated by PE is associated with an improvement of the mitochondrial function and an increase of BDNF in the cerebral levels and glial-cell-line-derived neurotrophic factors. According to Lau and coworkers, PE protects neurons and mitochondria but also increases synthesis of neurotrophic factors in the substantia nigra (nigrostriatal neurotrophic factors) in PD mice with moderate neurodegeneration [55].

**Table 1:** The recently published clinical data indicates that physical activity in PD is associated with reduce motion and balance impairments, as shown by the significant improvement in UPDRS III, functional mobility and muscle strength.

Study (ref)	Type of study	Type of excercise	Main outcomes
Leal et al. 2019 [120]	Clinical study on human (H&Y 1-3)	Low volume resistance training (6 months/twice weekly/30-40min/Interval break 1-2min).	The main result of this study shows that low RT volume improves the physical and functional capacity of older patients with PD. RT was efficient in promoting improvements in aerobic endurance, gait speed and balance.
Marusiak et al. 2019 [136]	Clinical study on human (H&Y 1.5-3)	Aerobic interval training (8 weeks/three times weekly/60min/Interval 8x5min, 2min <60rpm 3min >60rpm).	Moderate intensity aerobic interval training by individuals with PD improved psychomotor behaviors, as reflected by bimanual motor control, executive function, and neurological signs of PD.
Silva et al. 2019 [137]	Clinical study on human (H&Y 1-4)	Aquatic dual-task exercise (10 weeks/twice weekly/60min/group classes).	The aquatic exercise program improved the functional mobility, balance and gait of people with PD.
Rawson et al. 2019 [121]	Clinical study on human (H&Y 1-4)	Tango/Treadmill/ Stretching (12 weeks/twice weekly/60min/group classes).	No change in Quality of life scores all 3 group (PDQ-39). SMWT test Tango group improve the most. MDS-UPDRS-III all three group improve but Stretching significant.
Zhu et al. 2020 [138]	Clinical study on human (H&Y 1-3)	Tai Chi (12 weeks/group classes).	After 12 weeks of intervention, participants in both Tai Chi and routine exercise groups gained effects in UPDRS-III, BBS, PDQ-39, PDSS and HAMD compared to the baseline.
Sacheli et al. 2019 [122]	Clinical study on human (H&Y 1-3)	Aerobic exercise (3 months/three times weekly/cycling 40-60min/60%-80% VO2max).	The current study showed that after 3 months of aerobic exercise there was increased rTMS-evoked dopamine release in the caudate and greater activation of the ventral striatum in anticipation of reward. Dopaminergic changes are likely not the only explanation for the benefits of exercise in PD. Other mechanisms may include modulation of neuroinflammation, increases in glial and brain-derived trophic factors and cerebral blood flow.
Fleisher et al. 2020 [139]	Clinical study on human (H&Y 1-3)	Shotokan (10 weeks/twice weekly/60min/group classes).	We found a statistically and clinically significantly improve mention quality of life of 5.9 points on the PDQ-8. There were no changes in physical activity levels compared to baseline as measured by the IPAQ. We found no change in Functional Reach Test, and a small change in Tinetti Mobility Test.
Cherup et al. 2019 [140]	Clinical study on human (H&Y 1-3)	Strength and Power Training (12weeks/twice weekly/60min).	Subjects in both the PT and ST groups demonstrated significant improvements in muscular strength and power, both PRT programs appear helpful in addressing these neuromuscular performance variables.
Vieira de Moraes Filho et al. 2020 [141]	Clinical study on human (H&Y 1-3)	Progressive resistance training (9weeks/twice weekly/50-60min).	The present study highlights the PRT importance on the adjunctive PD treatment due to its efficacy in promoting improvements in the disease motor symptoms with a few weeks of intervention. The significant effects on bradykinesia without an increase in muscle strength, suggest that the intervention promoted neural enhancements in a short term to substantially improve the functional performance of trained individuals.

At another neurological level, aerobic training for PD rats (in sessions lasting 20-60 min) performed 5 days a week for 4 weeks can restore the expression of glial fibrillary acidic protein (GFAP) in the dorsal striatum. That indicates that astrocytes may play a role in producing the beneficial effects of PE in PD [56]. It was suggested that the reduction in GFAP expression is related to the reduced expansion of astrocytes, probably due to an increase in the synaptic function in the dorsal striatum induced by PE [56]. This observation also demonstrates the neuroprotective role of PE.

Furthermore, regular training of rats (from 5 to 23 months of age) for 18 months on a horizontal treadmill at a speed of 20 m/min for 20 min, twice a day, 5 days a week, also had a neuroprotective effect on the cerebellum [57], a part of the brain that is fundamentally involved in the command and control of movement and balance. They also reported that sedentary elderly rats had 11% fewer Purkinje cells (cerebellar efferents) and 9% smaller Purkinje cell soma volumes (*p*=0.02 for both) than exercising elderly rats, with the latter having the same number of Purkinje cells as young rats (5 months of age) [57].

As summarized in Table 1, recently published clinical data indicate that different kind of PE in PD is associated with significantly reduced motion and balance impairments, as shown by the significant improvement in UPDRS III, stride length, and gait velocity. Moreover, it is shown that regular PE may slow down the disease’s progression in patients suffering from PD and may reduce the risk of disease in healthy patients. However, despite many pieces of evidence from animal studies that PE has a beneficial effect in the PD model, there is still little direct evidence from clinical trials data (Table 1), which makes it difficult to propose the appropriate exercise and time duration, and intensity contributes to slowing down the disease progression.

### Diet and supplementation in PD

Nutritional habits and diet have a significant impact on the human body at all ages. However, older people, more often than younger people, require individual nutritional intervention because of past or chronic diseases or unhealthy eating habits. A healthy and proper diet is one factor that contributes to a healthy aging process, reducing or slowing down the course of many chronic diseases [58].

Appropriate diet therapy in ND is attributed to the name of a protective factor that reduces the risk of disease or mitigates the course of an already existing disease. On the other hand, a heterogeneous and nutrient-poor diet may disturb homeostasis, increasing the risk of disease or accelerating its course. A shortage of ingredients can be unfavorable, but contaminants found in food have an impact on our health [59]. Contaminants can enter food products from water, soil, air, or when spraying plants. A major problem is the presence of xenobiotics in the aquatic environment, which poses a severe threat to all organisms living in the aquatic environment. These compounds have neurotoxic, immunosuppressive, and hepatotoxic effects [60].

### Vitamins of group B and homocysteine

It is worth noting the B vitamins, which play an essential role in homocysteine (Hcy) metabolism. Hcy is an amino acid whose excess in the body may contribute to the progression of many chronic diseases, including ND. An adequate amount of Hcy in the range of 5 to 15 μmol/l ensures the nervous system’s proper functioning. A value exceeding the upper limit indicates hyperhomo-cysteinemia. Hyperhomocysteinemia, associated with reduced levels of methionine synthase (MS), activates the generation of reactive oxygen species (ROS) and limits the action of GPx. It also accelerates the death of dopaminergic neurons [61].

Hcy is a result of denitrification of methionine, an exogenous sulphuric amino acid consumed with food. The resulting Hcy can be subjected to two processes lowering its concentration in blood, consisting of remethylation (second transformation to non-toxic methionine) or transsulformation, where it is rebuilt to cysteine. Remethylation of Hcy into methionine is a process where the methyl group’s donor is folic acid, and the cofactor of MS is vitamin B12 (cobalamin) [62]. However, in the process of transsulfuration, the presence of the β-synthase cystathionine cofactor - an active form of vitamin B6 - pyridoxal phosphate - is important. Lack of these cofactors, together with reduced enzyme activity, cause hyperhomocysteinemia. Hcy, which has not undergone any of the two metabolic changes (to methionine or cysteine) is deposited in excessive blood concentrations, which is harmful to our body.

Factors that increase the build-up of higher serum Hcy concentrations include poor eating habits and abnormal lifestyles. It was also noted that smoking and increased consumption of products rich in methionine (dairy products, fish, meat), coffee, and alcohol can increase Hcy levels in the blood. It is worth mentioning that the use of L-DOPA increases the demand for B vitamins and folic acid, hence the importance of adequately planned nutrition in patients suffering from PD [63].

An increased level of Hcy also reduced muscular strength, this leads to a reduction in physical capabilities and thus increases the risk of falls and fractures. Bone is formed through calcium phosphate deposition onto the protein matrix composed mainly of collagen. It has been reported that hyperhomocysteinemia disrupts the collagen-cross links and impairs bone strength [64]. Therefore, to reduce Hcy in blood, it is recommended to follow a regular physical activity and the methylating diet, influencing DNA methylation, which regulates genes expression.

### Antioxidant vitamins

Many scientists focus on studying the effects of antioxidant vitamins on the development and progression of ND. These include vitamins C, E, and A as well as carotenoids, which take part in calming oxidative stress. Vitamins C and E have selected protective properties, protect nerve cells against the harmful effects of inflammation and oxidative stress. Kim and coworkers in 2017 observed decreased levels of α- and β-carotenes and lycopene in plasma, which decreased with the duration of PD. They suppose that this relationship may be related to the progression of ND [65]. In another study, it was observed that an increased supply of vitamin E in the diet lowered the risk of AD development, but its intake together with vitamin C and β-carotene in the form of dietary supplements did not bring such effects [66]. It was noted that a higher intake of vitamin E and β-carotene in the diet was associated with a lower risk of PD, and no correlation with vitamin C was found [67]. Therefore, it is advisable to include fresh fruit and vegetables in the diet, bearing in mind that excess fiber in the diet can significantly reduce the bioavailability of carotenoids and vitamins [68].

### Vitamin D

Vitamin D or calciferol is a term covering a group of fat-soluble steroidal organic compounds. There are two forms of vitamin D: ergocalciferol (vitamin D2), found in products of plant origin and yeasts, and cholecalciferol (vitamin D3), found in products of animal origin (fatty fish, fish oil). It is estimated that about 90% of the amount of vitamin D3 necessary for human is delivered from biosynthesis occurring in the skin under the influence of sunlight. Unfortunately, the majority of the population suffers from massive deficiencies of this vitamin. 7-dehydrocholesterol called provitamin D, present in each of us’s skin, under the influence of sunlight UVB, is converted into cholecalciferol. Next, cholecalciferol is transferred to the liver, where 25-hydrolase converts it into 25-hydroxycholecalciferol (25(OH)D3). The final synthesis to the active form of vitamin D occurs mainly in kidneys, where 25-hydroxycholecalciferol is enzymatically transformed into 1,25-dihydroxycholecalciferol (calcitriol) via the enzyme 1α-hydroxylase.

The classic effect of vitamin D is to regulate the calcium-phosphate balance. However, recent works show that vitamin D has a pleiotropic effect. For example, vitamin D has an anti-inflammatory effect by lowering the biosynthesis of pro-inflammatory interleukins, which tend to attack myelin sheaths of nerve cells, which is the cause of many ND. Brain function and vitamin D are closely related. Neurons synthesize active vitamin D, which regulates these cells’ proliferation in an autocrine manner [69]. This allows the brain to exhibit neuroplastic function through increased neuroprotection. Notably, active vitamin D regulates the expression of many proteins involved in 1) synaptic plasticity, counting drebrin, growth-associated protein 43 (GAP43), and connexin 43, 2) cytoskeleton maintenance, including neurofilament, tubulin, microtubule-associated protein-2 (MAP2), and 3) molecular transport of cell organelles, with creatine kinase, kinesin, RhoA, dynactin [70, 71]. In areas of the brain responsible for DA secretion, active vitamin D affects dopaminergic circuits’ function and connectivity [71].

Moreover, recent data from our laboratory showed that optimal concentration in serum 25(OH)D3 decreases oxidative stress, attenuates muscle atrophy, reduces pro-inflammatory markers, and elevates the biogenesis of mitochondria, as well as improves their function [72-74]. Vitamin D supplementation may also have a beneficial effect on aging-related neurodegeneration diseases retardation. Moreover, vitamin D deficiency, diagnosed early and adequately compensated by diet, sun exposure, and supplements, may minimize the risk or prevent the occurrence of many diseases such as PD, SM, AD, arterial hypertension, and atherosclerosis RA, or various types of cancer [75]. In addition, it has been reported that preoperative supplementation with vitamin D induced faster recovery in LBP patients after PLIF surgery [76].

### Mediterranean diet

The Mediterranean diet arouses a growing interest and its beneficial effects such as slow down the aging process, reduce the risk of illness, and slow down the progression of chronic diseases, including ND. The Mediterranean diet is a diet and healthy eating habits of Mediterranean countries, including Italians, Spaniards, Greeks, and French. In combine with physical activity, it may bring many health benefits. According to studies, the Mediterranean diet’s use significantly improves health conditions and reduces PD and AD incidence by 13% [77]. However, it is worth remembering about the pollution of water reservoirs, as food consumption such as fish and seafood is an essential source of exposure to various types of xenobiotics. Choose products from the least polluting waters, e. g. oceanic waters.

The Mediterranean diet is rich in fresh vegetables and fruits, cereal products, pulses, olive oil, nuts, cheese and yogurts, fish and seafood, while it is reduced in sweets, eggs, meat (especially beef), and animal fats. Results of FFQ in PD patients may suggest that a diet based on the components mentioned above was associated with lower PD progress rates [78] and a higher age of disease diagnosis [79]. Due to the high consumption of fruit and vegetables, the Mediterranean diet is rich in antioxidants. Despite ambiguous research results, it is recommended to consume products rich in antioxidants, as they may slow down the progression of many diseases [80].

### Fatty acids

Studies on the compound and effects of mono- and poly-unsaturated fatty acids (MUFA and PUFA, respectively) on chronic diseases are still ongoing. PUFA include omega-3 and omega-6 fatty acids. Omega-3 fatty acids, which can improve excitability of nerve cell membranes, increase the transmission of nerve impulses, and protection against damage caused by oxidative stress, are essential for the construction and proper functioning of the brain. Moreover, omega-3 fatty acids showed neuroprotective effects in animal models PD [81]. It was observed that people eating at least once a week fatty fish (herring, anchovy, salmon, mackerel) or crustaceans (providing eicosapentaenoic acid and docosahexaenoic acid) reduced the risk of dementia to 60%, including AD [82].

MUFA have a positive effect on the lipid profile, effectively lowering the ratio of LDL cholesterol to HDL cholesterol. Besides, olive oil has anti-cancer, antibacterial, antiviral, and anti-inflammatory effects, which may inhibit the progression of ND. For the most part, PUFA, including omega-3 fatty acids, are also a source of fat. It has been shown that eating fatty fish and seafood more than twice a week reduces the risk of developing AD by as much as 60% compared to people who have eaten it less often [83].

### Dairy products

It has been established that dairy products consumed in large quantities may be positively correlated with an increased risk of developing PD, especially in men [84], and faster disease progression [85]. Researchers are trying to determine which mechanism is responsible and which of the specific dairy products in the diet has the greatest impact on PD’s development and progression because the results in many studies are not unambiguous. It is believed that consuming large amounts of dairy products causes a decrease in uric acid [85], the decreased concentration of which was observed in patients with PD, and this level decreased with the progression of the disease [86].

### Selected nutraceuticals

Nutraceuticals are isolated single substances, pharmaceutical preparations having the character of a paralysis (combining drug and food features), which contain, among others, bioactive ingredients of natural origin, showing pro-health properties [87]. It has been shown that many ingredients of natural origin have a protective effect in the case of ND, and their action consists of modulating multistage signal pathways [88]. Nutraceuticals appear to be a beneficial option for disease prevention and treatment because of their natural origin in the food available and the potential absence of side effects [89]. Polyphenols are known for their antioxidant properties and may be used for ND. Their function is to scavenge ROS, and polyphenols may also chelate metal ions (copper, iron, and other heavy metals), which prevents the formation of hydroxyl radical. Products containing large amounts of polyphenols may increase neurogenesis [90]. There was a significant correlation between the lower risk of developing PD in men who consumed more flavonoids. Such a relationship was not noticed in women. It was also found that higher consumption of anthocyanins, quercetin, and epicatechin reduces the risk of PD progression within 20-22 years of observation [91].

Curcumin, a biologically active colorant obtained from Curcuma, a long oyster, is the main ingredient of the famous spice “curry” and shows a very wide spectrum of pro-healthy effects. In numerous studies, its antioxidant, anti-inflammatory, anticancer [92], and anticoagulant effects were found. It has been noted that the toxicity of ROS and α-synuclein [93] decreases after curcumin administration. In addition, it may increase or stabilize neurogenesis in adults [90].

Luteolin, which belongs to flavones and is found in broccoli, green pepper, celery, and thyme, has a strong neuroprotective effect. Effectively suppresses inflammatory processes of the body, reducing the synthesis of inflammatory mediators [94]. It also has a protective effect against induced cell apoptosis, which makes it suitable for preventing ND or slowing their progression [95].

Genistein, a natural compound of plant origin present in soya, is a phytoestrogen belonging to the group of isoflavones. Many studies have shown neurotrophic and neuroprotective effects of genistein. It has been proven to protect nerve cells against oxidative stress and toxic effects of β-amyloid [96]. In vivo studies proved its protective effect on dopaminergic neurons in PD model rats [97]. Moreover, genistein’s neuroprotective effects on apoptosis induced by 1-methyl-4-phenyl-1,2,3,6-tetrahydropyridine (MPTP) in black substance neurons [98] were demonstrated. However, the exact mechanism of action of genistein is still unclear. We found that genistein also plays anti-inflammatory effects (data not published yet). Tumor necrosis factor (TNF-α) significantly decreased in AD rats after 4 weeks of genistein’s treatment.

Quercetin is one of the most common flavonoids found in food products eaten daily. It shows antioxidant properties related to the ability to scavenge ROS, inhibition of oxidase activity, and anti-inflammatory effects. Besides, it has hepatoprotective and anti-aggregating properties [99]. It is believed that quercetin may be a protective factor in ND in which increased apoptosis of cells leads to AD, PD, Huntington’s disease (HD), Amyotrophic Lateral Sclerosis (ALS) [100], among others.

Resveratrol is a biologically active substance found mainly in red wine and dark chocolate. It is an effective antioxidant and antidepressant with neuroprotective effects. Studies prove that resveratrol may protect dopaminergic neurons in the intracerebral against damage [101]. Its properties, such as ROS capture and anti-inflammatory activity, are well documented [102].

Anthocyanins are a group of flavonic compounds, natural dyes (from orange to violet) found in many fruits and some vegetables. Studies have shown that a high intake of anthocyanins together with diet is associated with risk reduction and inhibition of PD progression [103]. It has been proven that delphinidin, which belongs to anthocyanins, e. g. in blackberries, is the most effective in neutralizing ROS. However, all anthocyanins have neuroprotective properties by inhibiting pro-inflammatory pathways and inducing antioxidative pathways [104].

Ginkgo Biloba is a popular plant that has been used for years to treat memory and concentration disorders. The unique therapeutic properties of ginkgo are distinguished by bilobalide, a biologically active substance belonging to the group of isoprenoids (terpenes). It has an anti-apoptotic effect on neurons’ death, which opens up new possibilities in the fight against ND. Besides, it protects neurons from the harmful effects of free radicals, stimulates the release of neuromediators in the brain, and inhibits their breakdown [105].

Ginseng (Panax Ginseng) is a perennial used for centuries in traditional Far Eastern medicine. It contains more than 30 biologically active compounds called ginsenosides, belonging to triterpene saponins [106]. It shows potent anti-inflammatory and antioxidant effects through its influence on nitric oxide production and antioxidant enzymes’ activity eliminating ROS. It shows neuroprotective properties in AD, PD, ALS and HD. In PD models, it prevents apoptosis and atrophy of dopaminergic neurons and has a positive effect on synucleinopathy [107].

Caffeine, one of the most popular stimulants, also has anti-inflammatory, antioxidant, and anti-apoptotic effects. Caffeine is believed to reduce PD’s risk if used at a dose of 3-5 mg/kg body weight [108]. One glass of freshly brewed coffee is about 90-100 mg of caffeine (or 40 mg/100 ml of coffee). However, it should be noted that excessive coffee intake leads to an increase in blood Hcy concentration, which increases the risk of developing PD and accelerates the already ongoing disease process. In 2016, Lee and his team [109] found out that coffee is not caffeine, but quercetin is a key neuroprotective ingredient.

Capsaicin is an organic compound from the alkaloid group, responsible for the hot and pungent taste of paprika. Studies indicate that a combination of capsaicin and resveratrol treatment has more neuroprotective benefits than a single treatment. Combining these compounds has a protective effect on nerve cells against the toxic effects of glutamate, reducing oxidative stress and apoptosis; both of these compounds may be useful in the treatment of ND [110].

Sulforaphane, an organic sulfur compound found in the highest concentration in broccoli and its sprouts, is a potent antioxidant. Many studies have shown its neuroprotective properties in different models of neurodegeneration. It can increase glutathione levels and enzymes dependent on it; thus, it may be useful in slowing PD progression and other ND by silencing oxidative stress and apoptosis [111].

In the present review, a lot of data from animal studies showed beneficial effects in slowing down the progression of neurodegenerative disorders. Although less evidence comes from selected clinical trials data, which are summarized in Table 2. However, it suggests that the use of dietary supplements may have a positive impact on functional tests and anatomical, and biochemical alterations in PD patients. Absolutely, changes occurring and related to a proper diet and use of supplements are associated with patients, which most of them suffer from obesity, depression and do not exercise at all. In a result, PD patients develop inflammation in their organism. Therefore, a proper diet with supplements and nutraceuticals for patients with PD seems to be the most effective and non-invasive therapy against the disease progression.

### Kynurenine metabolites and microbiota

Chronic systemic inflammation is widely accepted as a common risk factor for many diseases (diabetes, cancer, AD, PD, depressions, and others) and has further been observed in several patients suffering from fatigue and cognitive impairments [112]. Studies have shown that inflammatory stimuli, as they appear after acute exercise, activate the amino acid TRP breakdown within the KYN pathway [113]. The KYN pathway includes several enzymes that degrade 95% of the free TRP into diverse bioactive kynurenine pathway metabolites (KPMs), often referred to as kynurenines [114]. Exercise alters skeletal muscle kynurenine metabolism and may significantly change the levels of some of these metabolites both in the periphery and in the CNS [114, 115]. Recently, it was shown that PE leads to a peripheral breakdown of KYN to KYNA by inducing kynurenine aminotransferases (KATs) in muscle tissue [114]. In contrast to KYN, KYNA is unable to cross the blood-brain barrier (BBB).

**Figure 1.**
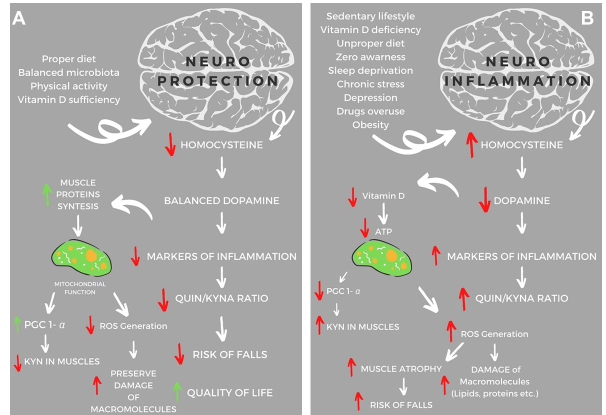
The scheme of neuroprotective and neuroinflammatory factors in PD’s. Based on animal and clinical trial studies [120, 138, 140, 146, 147, 151-153] the selected factors may influence on neuroprotection or neuroinflammation in reducing or worsening Parkinson’s decrease. (A) The proper diet combined with regular physical activity can lead to neuroprotection and consequently lowering the markers of inflammation and free radicals damage of macromolecules, improving the function of mitochondria as well as reducing the risk of osteoporosis, which is associated with decreasing risk of falls. (B) The improper diet and sedentary lifestyle may induce neuroinflammation. In consequences will be observed an increased Hcy level, a strong risk factor for osteoporotic fractures, mitochondrial dysfunction, ROS generation, damage of macromolecules, muscle atrophy and hence a deterioration in the quality-of-life patients. Abbreviation: KYN- kynurenine pathway, KYNA- kynurenine acid, QUIN- quinolinic acid, PGC-1α- peroxisome proliferator-activated receptor-gamma coactivator 1α, ROS-reactive oxygen species, and (indicators: ↓-decrease; ↑-increase).

The link between skeletal muscle, exercise, and KPMs came from the observation that trained muscle (or muscle with high peroxisome proliferator-activated receptor-gamma coactivator 1α (PGC-1α) levels) has very high levels of KAT enzymes [114-116]. This enhances peripheral KYN to KYNA conversion and prevents the accumulation of KYN in the brain, which is observed in particular mental health disorders such as stress-induced depression. Thus, PE reduces the neurotoxic KPMs, which was further described as cognitive dysfunction in neurological diseases [117, 118]. In mice, this detoxification mechanism was shown to reduce KYN-related excitotoxicity, neuroinflammation, and depressive-like behaviors [115]. Since this pathway’s activation is extremely dependent on PGC-1α, mice genetically engineered to lack expression of this coactivator in muscle show depressive-like behavior and a reduced capacity to catabolize KYN, even in the absence of any challenge. This highlighted skeletal muscle and exercise as novel regulators of TRP-KYN metabolism, with important systemic consequences [114, 115]. Schlittler and coauthors showed that humans participating in active endurance training display both an increased mRNA genes expression and protein content of the KAT. They also reported that following aerobic exercise, a transient flux of the KYN pathway occurs with a preferential increase in the KYNA concentration (63%) over QUIN (19%), leading to a decreased QUIN/KYNA ratio [116].

**Table 2:** The selected clinical trials related to the use of dietary supplements on functional tests and biological changes in PD patients.

Study (ref)	Type of study	Treatment	Main outcomes
Postuma et al. (2017) [142]	Randomized controlled trial	2 groups: 1st (n=57) 200 mg of caffeine twice daily in the morning and after lunch 2nd (n=61) 200 mg matching placebo treatment The dose was rising from 50 mg per week to meet the final dose at week 9. The supplementation lasted for 6 months.	No significant motor improvement (MDS-UPDRS III) in both groups.
Simon et al. (2015) [143]	Clinical Trial	groups: - low caffeine intake (≤300 mg daily, n=1288) - high caffeine intake (>300 mg daily, n=261) - based on questionnaire additionally, 10 mg of creatine per day (n=765) or matching placebo treatment (n=770) when ½ patients reached 5 years - the study has ended.	No difference in the disease progression (UPDRS) between groups with different caffeine intake. Increased disease progression (UPDRS) in group taking creatine combined with high caffeine intake.
Postuma et al. (2012) [144]	Randomized controlled trial	2 groups: 1st (n=30) 3 weeks 100 mg of caffeine twice daily upon awakening and immediately after lunch, next 3 weeks 200 mg of caffeine twice daily 2nd (n=31) matching placebo treatment.	Significant improvement in total UPDRS and UPDRS III total after 6 weeks in caffeine group. No difference in depression/PDQ-39.
Fan et al. (2018) [145]	Clinical Trial	10 male patients with idiopathic PD, 28 days of supplementation, 300 mg of blackcurrant anthocyanins twice daily.	Higher cGP concertation in CSF after supplementation - probably because of the uptake form plasma cGP. May be the evidence of oral availability and effective brain uptake.
Tamtaji et al. (2018) [146]	Randomized Controlled Trial	2 groups: 1st (n=20) 1000 mg omega-3 fatty acids plus 400 IU of vitamin E once daily for 12 weeks 2nd (n=20) matching placebo treatment request for no changes in physical activity during supplementation.	In omega-3 plus vitamin E supplementation group significant improvement in total UPDRS compared to placebo group (in UPDRS-part I, in other parts of UPDRS no). Omega-3 fatty acids and vitamin E co-supplementation for 12 weeks in PD patients significantly improved gene expression of TNF-α, PPAR-γ and LDLR and did not have effect on levels of IL-1 and IL-8.
Taghizadeh et al. (2017) [147]	Randomized Controlled Trial	2 groups: 1st (n=30) 1000 mg omega-3 fatty acids plus 400 IU of vitamin E once daily for 12 weeks 2nd (n=30) matching placebo treatment request for no changes in physical activity during supplementation 3-day food records and three physical activity records at week 0, 3, 9 and 12.	Omega-3 fatty acids and vitamin E co-supplementation in PD patients had positive effects on UPDRS, hs-CRP, TAC, GSH and markers of insulin metabolism and did not have effect on other markers of inflammation, oxidative stress, and lipid profiles.
Barichella et al. (2019) [148]	Randomized Controlled Trial	2 groups: 1st (n=75) twice daily whey protein-based oral liquid formula enriched with leucine and vitamin D for 30 days 2nd (n=75) standard diet without this muscle-targeted nutritional supplement. Each group received multidisciplinary intensive rehabilitation treatment.	Significant improvement in group receiving muscle-targeted nutritional supplement in 6-minute walking test distance, 4-meter gait speed course, Timed Up and Go test, skeletal muscle mass and skeletal muscle mass index.
Hiller et al. (2018) [149]	Randomized Controlled Trial	2 groups: 1st (n=24) placebo plus 1000 mg calcium carbonate 2nd (n=27) 13600 UI vitamin D plus calcium from Monday to Friday (68000 UI vitamin D per week). Supplementation lasts for 16 weeks.	The concentration of serum vitamin D in vitamin D group has risen and in placebo group was stable. High dose vitamin D supplementation does not improve balance.
Suzuki et al. (2013) [150]	Randomized Controlled Trial	2 groups: 1st (n=55) 1200 UI vitamin D daily for 12 months. 2nd (n=57) matching placebo treatment.	H&Y score worsen in placebo group and was unchanged in vitamin D group. Difference between groups was significant. The same situation in UPDRS part II scores. PDQ-39 score improved in vitamin D group and did not change in placebo group. VDR FokI TT genotype - significant and consistent response to vitamin D supplementation VDR FokI CT genotype - moderate response VDR FokI CC genotype - no significant response.
DiFrancisco-Donoghue et al. (2012) [151]	Randomized Controlled Trial	4 groups: 1st (n=9) vitamin supplementation 5 mg/day of folic acid, 2000 µg/day of cyanocobalamin (vitamin B12) and 25 mg/day of pyridoxal-5’-phosphate (vitamin B6) for 6 weeks 2nd (n=9) exercise group 40 min, twice weekly for 6 weeks 3rd (n=9) exercise + vitamin 4th (n=9) control - normal daily activities.	6 weeks of supplementation - lowered Hcy. 6 weeks of training - increased glutathione levels, improved strength and aerobic capacity. Exercise + vitamin - did not have greater effect of any of the measurements that have been made.
Nascimento et al. (2011) [152]	Controlled Clinical Trial	3 groups: 1st (n=17) PD patients with no physical activity for 6 months. 2nd (n=24) PD patients with aerobic physical activity, 3 times weekly for 60 minutes for 6 months. 3rd (n=19) healthy controls with no physical activity for 6 months.	Hcy levels were lower in PD who participated in aerobic physical activity compared with patients who did not. Also in PD patients who exercised Hcy levels were similar to healthy controls. PD patients who did not exercise received higher doses of L-dopa which can be connected with higher Hcy levels.
Lee et al. (2010) [153]	Randomized Controlled Trial	3 groups: 1st (n=14) Hcy-lowering therapy 5 mg folate, 500 µg mecobalamin 3 times daily 2nd (n=14) α-LA therapy 600 mg α-LA twice daily 3rd (n=13) control group. Every group received 500 mg calcium and 1000 IU vitamin D	Hcy-lowering therapy may prevent bone loss in PD patients taking levodopa.

Another interesting aspect is the role of gut microbiota in ND. In recent years have been reported a lot of data showing that exist a correlation between gut microbiota and neuromodulation. It is clear that intestinal bacteria have the potential to alter neurotransmitter activity, thus interacting with the host nervous system to regulate mental health. A recent systemic review illustrated how exercise contributes to the elevation of the gamma-aminobutyric acid level in the hypothalamus, which is associated with lowered resting blood pressure and heart rate. Moreover, DA was shown to be synthesized in the gastrointestinal tract during stressful situations. In general, gut microbiota was shown to facilitate the production and regulation of neurotransmitters and hormones, which influenced human well-being, mood, and subjective sense of neurodegeneration [119]. Thus, diet and lifestyle have a substantial impact on the development of PD in aging-related changes. For example, the proper diet and regular physical activity may slow down neurodegenerative changes (Fig. 1A), while the improper diet and sedentary lifestyle lead directly to PD development (Fig. 1B).

### Summary

**Figure 2.**
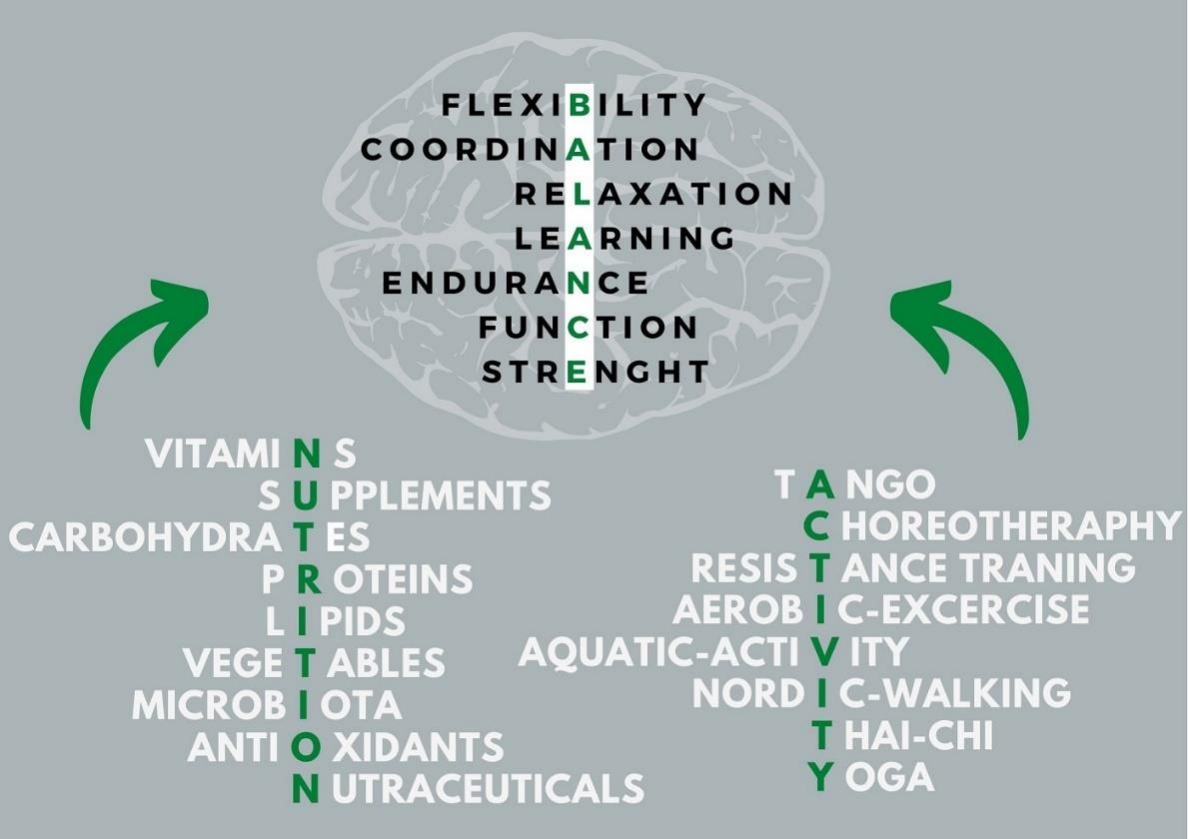
The impact of NUTRITION and ACTIVITY on BALANCE for people with Parkinson’s Disease.

Larger amounts of moderate-to-vigorous PE may slow down disease progression in PD patients [120-122]. It is known that PD patients expend 29% less energy than healthy subjects do, which lead to increased motor deficits and declines in daily activities [123]. PE is a nonpharmacological approach that is usually recommended for PD in order to slow down the deleterious effects of the disease [124]. Findings of some studies show that the beneficial effects of PE are cognitive performances or psychological domains (i.e., attentional capacities, depressive and anxiety symptoms, and mood state) [125]. Multicomponent training improves muscle strength, flexibility, postural balance, walking speed, mobility, functional capacity [126, 127], physical performance, and the activities of daily life [127], especially if the training program lasts more than 10 weeks [128]. Aerobic exercise, stretching, strength training, Qigong, and balance training improve motor function and in particular muscle strength, balance, and walking speed in PD subjects [129, 130]. These physical functioning effects may explain why the quality of life can be improved after only 6 weeks of training [131]. Jang and coworkers in 2018 showed that in PD rats endurance exercise improved mitochondria biogenesis and fusion [132]. Others also reported that, exercise enhanced the biogenesis of mitochondria by inducing the PGC-1α and improving antioxidant systems’ effectiveness [124, 132-134]. Based on the described above data and our knowledge, we suggest that NUTRITION combined with ACTIVITY will positively impact BALANCE not only in PD but also in other ND (Fig. 2).

### Conclusion

In terms of many researchs showing the positive effects of PE and proper diet in PD we have to focus to find the best therapy combining these two elements. On the other side, taking into consideration the individual variability in each human being, the past disease’s and the severity of the disease, it is challenging to determine one pattern that would be effective for everyone. Furthermore, we purpose to look for relationships between external and internal factors affecting the CNS. There are many indications that we are going in the right direction, going from exercise and diet to metabolic pathways leading to the nervous system.

The food and supplements associated with PD progression will probably change over time, with subsequent studies and evaluation of their results. Clinicians now have data to base a recommendation for healthy nutrition, and patients will probably be able to know that their daily choices can influence their progress.

Regular physical activity and a diet rich in the elements mentioned above may slow down the disease’s progression in patients suffering from PD and reduce the risk of disease in healthy patients. Although there is still little direct evidence from clinical trials data, related to appropriate exercise and diet, however, it is worth taking such efforts. Preparing individual therapy for a patient with PD in terms of exercise and diet is the most effective non-invasive therapy as of today. Further work on comprehensive therapy for patients should be complementary and include both.

Inflammatory (TNF-α, Interferon-gamma, IL-6, IL-17) and anti-inflammatory (IL-10, TGF-β) soluble factors as well as Tryptophan metabolites (TRP, KYN, KYNA, QUIN) that are known to be produced or secreted in response to exercise and that are further suspected to modify immune homeostasis and BBB function (MMP-2, MMP-9) through their inflammatory and anti-inflammatory properties. Studies have shown that regular physical activity positively affects motor and cognitive function in persons with ND. However, the potential mechanisms of exercise-induced changes in immune function remain theoretical constructs as no evidence is available that the achieved improvements will impact the person’s everyday life. Further, the influence of varying exercise intensities will provide more detailed recommendations for rehabilitative training programs.

### Future directions

In light of the current knowledge, combining proper diet with vitamin D supplementation may have a beneficial effect on muscle function, delaying aging-related ND development. Furthermore, it has been shown that reaching optimal serum concentration of vitamin D in LBP patients reduced markers of oxidative stress [72] and inflammation [74] as well as elevated biogenesis and function of mitochondria, and decreased skeletal muscle atrophy [73]. On the other side, the discovery of vitamin D receptor (VDR) and 1-alfa hydroxylase in skeletal muscle provided evidence showing the beneficial effects of exercise on proper muscle metabolism. Moreover, VDR may potentially regulate local control of vitamin D metabolism in the skeletal muscle. The proper synergistic diet, exercise, and vitamin D interaction towards muscle protein synthesis and mitochondrial function improvement may be manifested by mTOR and FOXO signaling influence on oxidative stress and immunological functions modulation. Further search for knowledge toward proper diet, exercise, and vitamin D impact on brain functions, chronic stress [135], and neuroprotection seem crucial as factors indirectly affect neuromodulation and neurodegeneration, especially in PD.

## References

[1] Huse DM, Schulman K, Orsini L, Castelli-Haley J, Kennedy S, Lenhart G (2005). Burden of illness in Parkinson’s disease. Mov Disord, 20:1449-1454. 16007641 10.1002/mds.20609

[2] Kerr GK, Worringham CJ, Cole MH, Lacherez PF, Wood JM, Silburn PA (2010). Predictors of future falls in Parkinson disease. Neurology, 75:116-124. 20574039 10.1212/WNL.0b013e3181e7b688

[3] Schaafsma JD, Giladi N, Balash Y, Bartels AL, Gurevich T, Hausdorff JM (2003). Gait dynamics in Parkinson’s disease: relationship to Parkinsonian features, falls and response to levodopa. J Neurol Sci, 212:47-53. 12809998 10.1016/s0022-510x(03)00104-7

[4] Lo RY, Tanner CM, Albers KB, Leimpeter AD, Fross RD, Bernstein AL, et al. (2009). Clinical features in early Parkinson disease and survival. Arch Neurol, 66:1353-1358. 19901166 10.1001/archneurol.2009.221

[5] Mazzoni P, Wexler NS (2009). Parallel explicit and implicit control of reaching. PLoS One, 4:e7557. 19847295 10.1371/journal.pone.0007557 PMC2760763

[6] Redgrave P, Rodriguez M, Smith Y, Rodriguez-Oroz MC, Lehericy S, Bergman H, et al. (2010). Goal-directed and habitual control in the basal ganglia: implications for Parkinson’s disease. Nat Rev Neurosci, 11:760-772. 20944662 10.1038/nrn2915 PMC3124757

[7] Wu T, Hallett M (2008). Neural correlates of dual task performance in patients with Parkinson’s disease. J Neurol Neurosurg Psychiatry, 79:760-766. 18006652 10.1136/jnnp.2007.126599

[8] Sutoo De, Akiyama K (2003). Regulation of brain function by exercise. Neurobiol Dis, 13:1-14. 12758062 10.1016/s0969-9961(03)00030-5

[9] Fox CM, Ramig LO, Ciucci MR, Sapir S, McFarland DH, Farley BG (2006). The science and practice of LSVT/LOUD: neural plasticity-principled approach to treating individuals with Parkinson disease and other neurological disorders. Semin Speech Lang, 27:283-299. 17117354 10.1055/s-2006-955118

[10] Devi SA, Kiran TR (2004). Regional responses in antioxidant system to exercise training and dietary vitamin E in aging rat brain. Neurobiol Aging, 25:501-508. 15013571 10.1016/S0197-4580(03)00112-X

[11] Alonso-Frech F, Sanahuja JJ, Rodriguez AM (2011). Exercise and physical therapy in early management of Parkinson disease. Neurologist, 17:S47-53. 22045326 10.1097/NRL.0b013e31823968ec

[12] Raefsky SM, Mattson MP (2017). Adaptive responses of neuronal mitochondria to bioenergetic challenges: Roles in neuroplasticity and disease resistance. Free Radic Biol Med, 102:203-216. 27908782 10.1016/j.freeradbiomed.2016.11.045 PMC5209274

[13] Winkler-Stuck K, Kirches E, Mawrin C, Dietzmann K, Lins H, Wallesch CW, et al. (2005). Re-evaluation of the dysfunction of mitochondrial respiratory chain in skeletal muscle of patients with Parkinson’s disease. J Neural Transm (Vienna), 112:499-518. 15340872 10.1007/s00702-004-0195-y

[14] Blin O, Desnuelle C, Rascol O, Borg M, Peyro Saint Paul H, Azulay JP, et al. (1994). Mitochondrial respiratory failure in skeletal muscle from patients with Parkinson’s disease and multiple system atrophy. J Neurol Sci, 125:95-101. 7964895 10.1016/0022-510x(94)90248-8

[15] Heischmann S, Gano LB, Quinn K, Liang L-P, Klepacki J, Christians U, et al. (2018). Regulation of kynurenine metabolism by a ketogenic diet. J Lipid Res, 59:958-966. 29605816 10.1194/jlr.M079251 PMC5983405

[16] Martin CR, Osadchiy V, Kalani A, Mayer EA (2018). The Brain-Gut-Microbiome Axis. Cell Mol Gastroenterol Hepatol, 6:133-148. 30023410 10.1016/j.jcmgh.2018.04.003 PMC6047317

[17] Baj A, Moro E, Bistoletti M, Orlandi V, Crema F, Giaroni C (2019). Glutamatergic Signaling Along The Microbiota-Gut-Brain Axis. Int J Mol Sci, 20:1482. 10.3390/ijms20061482 PMC6471396 30934533

[18] Agus A, Planchais J, Sokol H (2018). Gut Microbiota Regulation of Tryptophan Metabolism in Health and Disease. Cell Host Microbe, 23:716-724. 29902437 10.1016/j.chom.2018.05.003

[19] Sacks D, Baxter B, Campbell BCV, Carpenter JS, Cognard C, Dippel D, et al. (2018). Multisociety Consensus Quality Improvement Revised Consensus Statement for Endovascular Therapy of Acute Ischemic Stroke. Int J Stroke, 13:612-632. 29786478 10.1177/1747493018778713

[20] Ashe MC, Miller WC, Eng JJ, Noreau L, Physical A, Chronic Conditions Research T (2009). Older adults, chronic disease and leisure-time physical activity. Gerontology, 55:64-72. 18566534 10.1159/000141518 PMC3167824

[21] Canning CG, Sherrington C, Lord SR, Close JCT, Heritier S, Heller GZ, et al. (2015). Exercise for falls prevention in Parkinson disease: a randomized controlled trial. Neurology, 84:304-312. 25552576 10.1212/WNL.0000000000001155 PMC4335992

[22] Ellis T, Rochester L (2018). Mobilizing Parkinson’s Disease: The Future of Exercise. J Parkinsons Dis, 8:S95-S100. 30584167 10.3233/JPD-181489 PMC6311359

[23] Xu X, Fu Z, Le W (2019). Exercise and Parkinson’s disease. Int Rev Neurobiol, 147:45-74. 31607362 10.1016/bs.irn.2019.06.003

[24] Mak MK, Wong-Yu IS, Shen X, Chung CL (2017). Long-term effects of exercise and physical therapy in people with Parkinson disease. Nat Rev Neurol, 13:689-703. 29027544 10.1038/nrneurol.2017.128

[25] Mehrholz J, Friis R, Kugler J, Twork S, Storch A, Pohl M (2010). Treadmill training for patients with Parkinson’s disease. Cochrane Database Syst Rev, 1:CD007830. 10.1002/14651858.CD007830.pub2 20091652

[26] Silveira CRA, Roy EA, Intzandt BN, Almeida QJ (2018). Aerobic exercise is more effective than goal-based exercise for the treatment of cognition in Parkinson’s disease. Brain Cogn, 122:1-8. 29331916 10.1016/j.bandc.2018.01.002

[27] Altmann LJP, Stegemoller E, Hazamy AA, Wilson JP, Bowers D, Okun MS, et al. (2016). Aerobic Exercise Improves Mood, Cognition, and Language Function in Parkinson’s Disease: Results of a Controlled Study. J Int Neuropsychol Soc, 22:878-889. 27655232 10.1017/S135561771600076X

[28] van Eijkeren FJM, Reijmers RSJ, Kleinveld MJ, Minten A, Bruggen JPT, Bloem BR (2008). Nordic walking improves mobility in Parkinson’s disease. Mov Disord, 23:2239-2243. 18816697 10.1002/mds.22293

[29] Mak MKY, Wong-Yu ISK (2019). Exercise for Parkinson’s disease. Int Rev Neurobiol, 147:1-44. 31607351 10.1016/bs.irn.2019.06.001

[30] Warlop T, Detrembleur C, Buxes Lopez M, Stoquart G, Lejeune T, Jeanjean A (2017). Does Nordic Walking restore the temporal organization of gait variability in Parkinson’s disease? J Neuroeng Rehabil, 14:17. 28222810 10.1186/s12984-017-0226-1 PMC5320697

[31] Franzoni LT, Monteiro EP, Oliveira HB, da Rosa RG, Costa RR, Rieder C, et al. (2018). A 9-Week Nordic and Free Walking Improve Postural Balance in Parkinson’s Disease. Sports Med Int Open, 2:E28-E34. 30539114 10.1055/s-0043-124757 PMC6225959

[32] Monteiro EP, Franzoni LT, Cubillos DM, de Oliveira Fagundes A, Carvalho AR, Oliveira HB, et al. (2017). Effects of Nordic walking training on functional parameters in Parkinson’s disease: a randomized controlled clinical trial. Scand J Med Sci Sports, 27:351-358. 26833853 10.1111/sms.12652

[33] Bang D-H, Shin W-S (2017). Effects of an intensive Nordic walking intervention on the balance function and walking ability of individuals with Parkinson’s disease: a randomized controlled pilot trial. Aging Clin Exp Res, 29:993-999. 27798812 10.1007/s40520-016-0648-9

[34] Brienesse LA, Emerson MN (2013). Effects of resistance training for people with Parkinson’s disease: a systematic review. J Am Med Dir Assoc, 14:236-241. 23318666 10.1016/j.jamda.2012.11.012

[35] Ferreira RM, Alves WMGdC, de Lima TA, Alves TGG, Alves Filho PAM, Pimentel CP, et al. (2018). The effect of resistance training on the anxiety symptoms and quality of life in elderly people with Parkinson’s disease: a randomized controlled trial. Arq Neuropsiquiatr, 76:499-506. 30231121 10.1590/0004-282X20180071

[36] Silva-Batista C, Corcos DM, Barroso R, David FJ, Kanegusuku H, Forjaz C, et al. (2017). Instability Resistance Training Improves Neuromuscular Outcome in Parkinson’s Disease. Med Sci Sports Exerc, 49:652-660. 27851668 10.1249/MSS.0000000000001159

[37] Schlenstedt C, Paschen S, Kruse A, Raethjen J, Weisser B, Deuschl G (2015). Resistance versus Balance Training to Improve Postural Control in Parkinson’s Disease: A Randomized Rater Blinded Controlled Study. PLoS One, 10:e0140584. 26501562 10.1371/journal.pone.0140584 PMC4621054

[38] Earhart GM (2009). Dance as therapy for individuals with Parkinson disease. Eur J Phys Rehabil Med, 45:231-238. 19532110 PMC2780534

[39] Hackney ME, Earhart GM (2009). Effects of dance on movement control in Parkinson’s disease: a comparison of Argentine tango and American ballroom. J Rehabil Med, 41:475-481. 19479161 10.2340/16501977-0362 PMC2688709

[40] Kalyani HHN, Sullivan KA, Moyle G, Brauer S, Jeffrey ER, Kerr GK (2019). Impacts of dance on cognition, psychological symptoms and quality of life in Parkinson’s disease. NeuroRehabilitation, 45:273-283. 31561398 10.3233/NRE-192788

[41] de Natale ER, Paulus KS, Aiello E, Sanna B, Manca A, Sotgiu G, et al. (2017). Dance therapy improves motor and cognitive functions in patients with Parkinson’s disease. NeuroRehabilitation, 40:141-144. 27814308 10.3233/NRE-161399

[42] Solla P, Cugusi L, Bertoli M, Cereatti A, Della Croce U, Pani D, et al. (2019). Sardinian Folk Dance for Individuals with Parkinson’s Disease: A Randomized Controlled Pilot Trial. J Altern Complement Med, 25:305-316. 30624952 10.1089/acm.2018.0413

[43] Pereira APS, Marinho V, Gupta D, Magalhaes F, Ayres C, Teixeira S (2019). Music Therapy and Dance as Gait Rehabilitation in Patients With Parkinson Disease: A Review of Evidence. J Geriatr Psychiatry Neurol, 32:49-56. 30558462 10.1177/0891988718819858

[44] Zhang Q, Hu J, Wei L, Jia Y, Jin Y (2019). Effects of dance therapy on cognitive and mood symptoms in people with Parkinson’s disease: A systematic review and meta-analysis. Complement Ther Clin Pract, 36:12-17. 31383428 10.1016/j.ctcp.2019.04.005

[45] Klein PJ, Rivers L (2006). Taiji for individuals with Parkinson disease and their support partners: a program evaluation. J Neurol Phys Ther, 30:22-27. 16630368 10.1097/01.npt.0000282146.18446.f1

[46] Amano S, Nocera JR, Vallabhajosula S, Juncos JL, Gregor RJ, Waddell DE, et al. (2013). The effect of Tai Chi exercise on gait initiation and gait performance in persons with Parkinson’s disease. Parkinsonism Relat Disord, 19:955-960. 23835431 10.1016/j.parkreldis.2013.06.007 PMC3825828

[47] Li F, Harmer P, Fitzgerald K, Eckstrom E, Stock R, Galver J, et al. (2012). Tai chi and postural stability in patients with Parkinson’s disease. N Engl J Med, 366:511-519. 22316445 10.1056/NEJMoa1107911 PMC3285459

[48] Hackney ME, Earhart GM (2008). Tai Chi improves balance and mobility in people with Parkinson disease. Gait Posture, 28:456-460. 18378456 10.1016/j.gaitpost.2008.02.005 PMC2552999

[49] Li Q, Liu J, Dai F, Dai F (2020). Tai Chi versus routine exercise in patients with early- or mild-stage Parkinson’s disease: a retrospective cohort analysis. Braz J Med Biol Res, 53:e9171. 32049101 10.1590/1414-431X20199171 PMC7013627

[50] Paillard T, Rolland Y, de Souto Barreto P (2015). Protective Effects of Physical Exercise in Alzheimer’s Disease and Parkinson’s Disease: A Narrative Review. J Clin Neurol, 11:212-219. 26174783 10.3988/jcn.2015.11.3.212 PMC4507374

[51] Zigmond MJ, Cameron JL, Hoffer BJ, Smeyne RJ (2012). Neurorestoration by physical exercise: moving forward. Parkinsonism Relat Disord, 18 Suppl 1:S147-150. 22166417 10.1016/S1353-8020(11)70046-3

[52] Speelman AD, van de Warrenburg BP, van Nimwegen M, Petzinger GM, Munneke M, Bloem BR (2011). How might physical activity benefit patients with Parkinson disease? Nat Rev Neurol, 7:528-534. 21750523 10.1038/nrneurol.2011.107

[53] Wu S-Y, Wang T-F, Yu L, Jen CJ, Chuang J-I, Wu F-S, et al. (2011). Running exercise protects the substantia nigra dopaminergic neurons against inflammation-induced degeneration via the activation of BDNF signaling pathway. Brain Behav Immun, 25:135-146. 20851176 10.1016/j.bbi.2010.09.006

[54] Petzinger GM, Fisher BE, Van Leeuwen J-E, Vukovic M, Akopian G, Meshul CK, et al. (2010). Enhancing neuroplasticity in the basal ganglia: the role of exercise in Parkinson’s disease. Mov Disord, 25 Suppl 1:S141-145. 20187247 10.1002/mds.22782 PMC4111643

[55] Lau Y-S, Patki G, Das-Panja K, Le W-D, Ahmad SO (2011). Neuroprotective effects and mechanisms of exercise in a chronic mouse model of Parkinson’s disease with moderate neurodegeneration. Eur J Neurosci, 33:1264-1274. 21375602 10.1111/j.1460-9568.2011.07626.x PMC3079264

[56] Dutra MF, Jaeger M, Ilha J, Kalil-Gaspar PI, Marcuzzo S, Achaval M (2012). Exercise improves motor deficits and alters striatal GFAP expression in a 6-OHDA-induced rat model of Parkinson’s disease. Neurol Sci, 33:1137-1144. 22231471 10.1007/s10072-011-0925-5

[57] Larsen JO, Skalicky M, Viidik A (2000). Does long-term physical exercise counteract age-related Purkinje cell loss? A stereological study of rat cerebellum. J Comp Neurol, 428:213-222. 11064362

[58] Wyka J (2012). [Nutritional factors in prevention of Alzheimer’s disease]. Czynniki zywieniowe w zapobieganiu chorobie alzheimera. Rocz Panstw Zakl Hig, 63:135-140. 22928359

[59] Przysławski J, Stelmach M (2008). Rola składników odżywczych w łagodzeniu objawów choroby Alzheimera. Żyw Człow Metab, 35:332-339.

[60] Tiano L, Fedeli D, Santoni G, Davies I, Falcioni G (2003). Effect of tributyltin on trout blood cells: changes in mitochondrial morphology and functionality. Biochim Biophys Acta, 1640:105-112. 12729919 10.1016/s0167-4889(03)00025-9

[61] de Lau LML, Koudstaal PJ, Witteman JCM, Hofman A, Breteler MMB (2006). Dietary folate, vitamin B12, and vitamin B6 and the risk of Parkinson disease. Neurology, 67:315-318. 16864826 10.1212/01.wnl.0000225050.57553.6d

[62] Fudala M, Brola W, Przybylski W, Czernicki J (2008). Is research in homocysteine and cyanocobalamine levels likely to become the key to diagnosing and treating Alzheimer diseases? Med Stud, 10:53-58.

[63] Refsum H, Smith AD, Ueland PM, Nexo E, Clarke R, McPartlin J, et al. (2004). Facts and recommendations about total homocysteine determinations: an expert opinion. Clin Chem, 50:3-32. 14709635 10.1373/clinchem.2003.021634

[64] Ao M, Inuiya N, Ohta J, Kurose S, Takaoka H, Abe Y, et al. (2019). Relationship between Homocysteine, Folate, Vitamin B12 and Physical Performance in the Institutionalized Elderly. J Nutr Sci Vitaminol (Tokyo), 65:1-7. 30814404 10.3177/jnsv.65.1

[65] Kim JH, Hwang J, Shim E, Chung E-J, Jang SH, Koh S-B (2017). Association of serum carotenoid, retinol, and tocopherol concentrations with the progression of Parkinson’s Disease. Nutr Res Pract, 11:114-120. 28386384 10.4162/nrp.2017.11.2.114 PMC5376529

[66] Morris MC, Evans DA, Bienias JL, Tangney CC, Bennett DA, Aggarwal N, et al. (2002). Dietary intake of antioxidant nutrients and the risk of incident Alzheimer disease in a biracial community study. Jama, 287:3230-3237. 12076219 10.1001/jama.287.24.3230

[67] Yang F, Wolk A, Hakansson N, Pedersen NL, Wirdefeldt K (2017). Dietary antioxidants and risk of Parkinson’s disease in two population-based cohorts. Mov Disord, 32:1631-1636. 28881039 10.1002/mds.27120 PMC5698752

[68] Bjelakovic G, Nikolova D, Gluud LL, Simonetti RG, Gluud C (2007). Mortality in randomized trials of antioxidant supplements for primary and secondary prevention: systematic review and meta-analysis. Jama, 297:842-857. 17327526 10.1001/jama.297.8.842

[69] Eyles DW, Smith S, Kinobe R, Hewison M, McGrath JJ (2005). Distribution of the vitamin D receptor and 1 alpha-hydroxylase in human brain. J Chem Neuroanat, 29:21-30. 15589699 10.1016/j.jchemneu.2004.08.006

[70] Eyles D, Almeras L, Benech P, Patatian A, Mackay-Sim A, McGrath J, et al. (2007). Developmental vitamin D deficiency alters the expression of genes encoding mitochondrial, cytoskeletal and synaptic proteins in the adult rat brain. J Steroid Biochem Mol Biol, 103:538-545. 17293106 10.1016/j.jsbmb.2006.12.096

[71] Trinko JR, Land BB, Solecki WB, Wickham RJ, Tellez LA, Maldonado-Aviles J, et al. (2016). Vitamin D3: A Role in Dopamine Circuit Regulation, Diet-Induced Obesity, and Drug Consumption. eNeuro, 3. 10.1523/ENEURO.0122-15.2016 PMC4875352 27257625

[72] Dzik K, Skrobot W, Flis DJ, Karnia M, Libionka W, Kloc W, et al. (2018). Vitamin D supplementation attenuates oxidative stress in paraspinal skeletal muscles in patients with low back pain. Eur J Appl Physiol, 118:143-151. 29143122 10.1007/s00421-017-3755-1

[73] Dzik KP, Skrobot W, Kaczor KB, Flis DJ, Karnia MJ, Libionka W, et al. (2019). Vitamin D Deficiency Is Associated with Muscle Atrophy and Reduced Mitochondrial Function in Patients with Chronic Low Back Pain. Oxid Med Cell Longev, 2019:6835341. 31281588 10.1155/2019/6835341 PMC6589343

[74] Krasowska K, Skrobot W, Liedtke E, Sawicki P, Flis DJ, Dzik KP, et al. (2019). The Preoperative Supplementation With Vitamin D Attenuated Pain Intensity and Reduced the Level of Pro-inflammatory Markers in Patients After Posterior Lumbar Interbody Fusion. Front Pharmacol, 10:527. 31191300 10.3389/fphar.2019.00527 PMC6539202

[75] Charoenngam N, Holick MF (2020). Immunologic Effects of Vitamin D on Human Health and Disease. Nutrients, 12:2097. 10.3390/nu12072097 PMC7400911 32679784

[76] Skrobot W, Liedtke E, Krasowska K, Dzik KP, Flis DJ, Samoraj-Dereszkiewicz A, et al. (2019). Early Rehabilitation Program and Vitamin D Supplementation Improves Sensitivity of Balance and the Postural Control in Patients after Posterior Lumbar Interbody Fusion: A Randomized Trial. Nutrients, 11:2202. 10.3390/nu11092202 PMC6769962 31547377

[77] Sofi F, Cesari F, Abbate R, Gensini GF, Casini A (2008). Adherence to Mediterranean diet and health status: meta-analysis. BMJ, 337:a1344. 18786971 10.1136/bmj.a1344 PMC2533524

[78] Mischley LK, Lau RC, Bennett RD (2017). Role of Diet and Nutritional Supplements in Parkinson’s Disease Progression. Oxid Med Cell Longev, 2017:6405278. 29081890 10.1155/2017/6405278 PMC5610862

[79] Alcalay RN, Gu Y, Mejia-Santana H, Cote L, Marder KS, Scarmeas N (2012). The association between Mediterranean diet adherence and Parkinson’s disease. Mov Disord, 27:771-774. 22314772 10.1002/mds.24918 PMC3349773

[80] Sofi F, Macchi C, Abbate R, Gensini GF, Casini A (2013). Mediterranean diet and health. Biofactors, 39:335-342. 23553669 10.1002/biof.1096

[81] Bousquet M, Gue K, Emond V, Julien P, Kang JX, Cicchetti F, et al. (2011). Transgenic conversion of omega-6 into omega-3 fatty acids in a mouse model of Parkinson’s disease. J Lipid Res, 52:263-271. 21115966 10.1194/jlr.M011692 PMC3023546

[82] Barberger-Gateau P, Letenneur L, Deschamps V, Peres K, Dartigues J-F, Renaud S (2002). Fish, meat, and risk of dementia: cohort study. BMJ, 325:932-933. 12399342 10.1136/bmj.325.7370.932 PMC130057

[83] Dochniak M, Ekiert K (2015). Nutrition in Prevention and Treatment of Alzheimer’s and Parkinson’s Diseases. Piel Zdr Publ, 5:199-208.

[84] Jiang W, Ju C, Jiang H, Zhang D (2014). Dairy foods intake and risk of Parkinson’s disease: a dose-response meta-analysis of prospective cohort studies. Eur J Epidemiol, 29:613-619. 24894826 10.1007/s10654-014-9921-4

[85] Dalbeth N, Ames R, Gamble GD, Horne A, Wong S, Kuhn-Sherlock B, et al. (2012). Effects of skim milk powder enriched with glycomacropeptide and G600 milk fat extract on frequency of gout flares: a proof-of-concept randomised controlled trial. Ann Rheum Dis, 71:929-934. 22275296 10.1136/annrheumdis-2011-200156

[86] Wen M, Zhou B, Chen Y-H, Ma Z-L, Gou Y, Zhang C-L, et al. (2017). Serum uric acid levels in patients with Parkinson’s disease: A meta-analysis. PLoS One, 12:e0173731. 28319195 10.1371/journal.pone.0173731 PMC5358777

[87] Bianchi VE, Herrera PF, Laura R (2019). Effect of nutrition on neurodegenerative diseases. A systematic review. Nutr Neurosci. 10.1080/1028415X.2019.1681088 31684843

[88] Kannappan R, Gupta SC, Kim JH, Reuter S, Aggarwal BB (2011). Neuroprotection by spice-derived nutraceuticals: you are what you eat! Mol Neurobiol, 44:142-159. 21360003 10.1007/s12035-011-8168-2 PMC3183139

[89] Hang L, Basil AH, Lim K-L (2016). Nutraceuticals in Parkinson’s Disease. Neuromolecular Med, 18:306-321. 27147525 10.1007/s12017-016-8398-6 PMC4983279

[90] Poulose SM, Miller MG, Scott T, Shukitt-Hale B (2017). Nutritional Factors Affecting Adult Neurogenesis and Cognitive Function. Adv Nutr, 8:804-811. 29141966 10.3945/an.117.016261 PMC5683005

[91] Gao X, Chen H, Fung TT, Logroscino G, Schwarzschild MA, Hu FB, et al. (2007). Prospective study of dietary pattern and risk of Parkinson disease. Am J Clin Nutr, 86:1486-1494. 17991663 10.1093/ajcn/86.5.1486 PMC2225168

[92] Anand P, Sundaram C, Jhurani S, Kunnumakkara AB, Aggarwal BB (2008). Curcumin and cancer: an "old-age" disease with an "age-old" solution. Cancer Lett, 267:133-164. 18462866 10.1016/j.canlet.2008.03.025

[93] Liu Z, Yu Y, Li X, Ross CA, Smith WW (2011). Curcumin protects against A53T alpha-synuclein-induced toxicity in a PC12 inducible cell model for Parkinsonism. Pharmacol Res, 63:439-444. 21237271 10.1016/j.phrs.2011.01.004

[94] Nabavi SF, Braidy N, Gortzi O, Sobarzo-Sanchez E, Daglia M, Skalicka-Wozniak K, et al. (2015). Luteolin as an anti-inflammatory and neuroprotective agent: A brief review. Brain Res Bull, 119:1-11. 26361743 10.1016/j.brainresbull.2015.09.002

[95] Guo D-J, Li F, Yu PH-F, Chan S-W (2013). Neuroprotective effects of luteolin against apoptosis induced by 6-hydroxydopamine on rat pheochromocytoma PC12 cells. Pharm Biol, 51:190-196. 23035972 10.3109/13880209.2012.716852

[96] Valles SL, Borras C, Gambini J, Furriol J, Ortega A, Sastre J, et al. (2008). Oestradiol or genistein rescues neurons from amyloid beta-induced cell death by inhibiting activation of p38. Aging Cell, 7:112-118. 18031570 10.1111/j.1474-9726.2007.00356.x

[97] Baluchnejadmojarad T, Roghani M, Nadoushan MRJ, Bagheri M (2009). Neuroprotective effect of genistein in 6-hydroxydopamine hemi-parkinsonian rat model. Phytother Res, 23:132-135. 18693302 10.1002/ptr.2564

[98] Liu L-X, Chen W-F, Xie J-X, Wong M-S (2008). Neuroprotective effects of genistein on dopaminergic neurons in the mice model of Parkinson’s disease. Neurosci Res, 60:156-161. 18054104 10.1016/j.neures.2007.10.005

[99] Williamson G, Manach C (2005). Bioavailability and bioefficacy of polyphenols in humans. II. Review of 93 intervention studies. Am J Clin Nutr, 81:243S-255S. 15640487 10.1093/ajcn/81.1.243S

[100] Kobylinska A, Janas KM (2015). [Health--promoting effect of quercetin in human diet]. Postepy Hig Med Dosw, 69:51-62. 10.5604/17322693.1135423 25589713

[101] Okawara M, Katsuki H, Kurimoto E, Shibata H, Kume T, Akaike A (2007). Resveratrol protects dopaminergic neurons in midbrain slice culture from multiple insults. Biochem Pharmacol, 73:550-560. 17147953 10.1016/j.bcp.2006.11.003

[102] Wood LG, Wark PAB, Garg ML (2010). Antioxidant and anti-inflammatory effects of resveratrol in airway disease. Antioxid Redox Signal, 13:1535-1548. 20214495 10.1089/ars.2009.3064

[103] Gao X, Cassidy A, Schwarzschild MA, Rimm EB, Ascherio A (2012). Habitual intake of dietary flavonoids and risk of Parkinson disease. Neurology, 78:1138-1145. 22491871 10.1212/WNL.0b013e31824f7fc4 PMC3320056

[104] de Pascual-Teresa S (2014). Molecular mechanisms involved in the cardiovascular and neuroprotective effects of anthocyanins. Arch Biochem Biophys, 559:68-74. 24791600 10.1016/j.abb.2014.04.012

[105] Tanaka K, Galduroz RFS, Gobbi LTB, Galduroz JCF (2013). Ginkgo biloba extract in an animal model of Parkinson’s disease: a systematic review. Curr Neuropharmacol, 11:430-435. 24381532 10.2174/1570159X11311040006 PMC3744905

[106] Rotblatt MD (1999). Herbal medicine: a practical guide to safety and quality assurance. West J Med, 171:172-175. 10560292 PMC1305803

[107] Van Kampen JM, Baranowski DB, Shaw CA, Kay DG (2014). Panax ginseng is neuroprotective in a novel progressive model of Parkinson’s disease. Exp Gerontol, 50:95-105. 24316034 10.1016/j.exger.2013.11.012

[108] Kolahdouzan M, Hamadeh MJ (2017). The neuroprotective effects of caffeine in neurodegenerative diseases. CNS Neurosci Ther, 23:272-290. 28317317 10.1111/cns.12684 PMC6492672

[109] Lee M, McGeer EG, McGeer PL (2016). Quercetin, not caffeine, is a major neuroprotective component in coffee. Neurobiol Aging, 46:113-123. 27479153 10.1016/j.neurobiolaging.2016.06.015

[110] Lee JG, Yon JM, Lin C, Jung AY, Jung KY, Nam SY (2012). Combined treatment with capsaicin and resveratrol enhances neuroprotection against glutamate-induced toxicity in mouse cerebral cortical neurons. Food Chem Toxicol, 50:3877-3885. 22943972 10.1016/j.fct.2012.08.040

[111] Morroni F, Tarozzi A, Sita G, Bolondi C, Zolezzi Moraga JM, Cantelli-Forti G, et al. (2013). Neuroprotective effect of sulforaphane in 6-hydroxydopamine-lesioned mouse model of Parkinson’s disease. Neurotoxicology, 36:63-71. 23518299 10.1016/j.neuro.2013.03.004

[112] Hanken K, Eling P, Hildebrandt H (2014). The representation of inflammatory signals in the brain - a model for subjective fatigue in multiple sclerosis. Front Neurol, 5:264. 25566171 10.3389/fneur.2014.00264 PMC4263099

[113] Fallarino F, Grohmann U, Puccetti P (2012). Indoleamine 2,3-dioxygenase: from catalyst to signaling function. Eur J Immunol, 42:1932-1937. 22865044 10.1002/eji.201242572

[114] Agudelo LZ, Femenia T, Orhan F, Porsmyr-Palmertz M, Goiny M, Martinez-Redondo V, et al. (2014). Skeletal muscle PGC-1alpha1 modulates kynurenine metabolism and mediates resilience to stress-induced depression. Cell, 159:33-45. 25259918 10.1016/j.cell.2014.07.051

[115] Martin KS, Azzolini M, Lira Ruas J (2020). The kynurenine connection: how exercise shifts muscle tryptophan metabolism and affects energy homeostasis, the immune system, and the brain. Cell physiol, 318:C818-C830. 10.1152/ajpcell.00580.2019 32208989

[116] Schlittler M, Goiny M, Agudelo LZ, Venckunas T, Brazaitis M, Skurvydas A, et al. (2016). Endurance exercise increases skeletal muscle kynurenine aminotransferases and plasma kynurenic acid in humans. Am J Physiol Cell Physiol, 310:C836-840. 27030575 10.1152/ajpcell.00053.2016

[117] Pocivavsek A, Wu HQ, Potter MC, Elmer GI, Pellicciari R, Schwarcz R (2011). Fluctuations in endogenous kynurenic acid control hippocampal glutamate and memory. Neuropsychopharmacology, 36:2357-2367. 21796108 10.1038/npp.2011.127 PMC3176574

[118] Stone TW, Darlington LG (2013). The kynurenine pathway as a therapeutic target in cognitive and neurodegenerative disorders. Br J Pharmacol, 169:1211-1227. 23647169 10.1111/bph.12230 PMC3831703

[119] Clark A, Mach N (2016). Exercise-induced stress behavior, gut-microbiota-brain axis and diet: a systematic review for athletes. J Sports Med Phys Fitness, 13:43. 10.1186/s12970-016-0155-6 PMC5121944 27924137

[120] Leal LC, Abrahin O, Rodrigues RP, da Silva MC, Araujo AP, de Sousa EC, et al. (2019). Low-volume resistance training improves the functional capacity of older individuals with Parkinson’s disease. Geriatr Gerontol Int, 19:635-640. 31037806 10.1111/ggi.13682

[121] Rawson KS, McNeely ME, Duncan RP, Pickett KA, Perlmutter JS, Earhart GM (2019). Exercise and Parkinson Disease: Comparing Tango, Treadmill, and Stretching. J Neurol Phys Ther, 43:26-32. 30531383 10.1097/NPT.0000000000000245 PMC6294320

[122] Sacheli MA, Neva JL, Lakhani B, Murray DK, Vafai N, Shahinfard E, et al. (2019). Exercise increases caudate dopamine release and ventral striatal activation in Parkinson’s disease. Mov Disord, 34:1891-1900. 31584222 10.1002/mds.27865

[123] Ahlskog JE (2011). Does vigorous exercise have a neuroprotective effect in Parkinson disease? Neurology, 77:288-294. 21768599 10.1212/WNL.0b013e318225ab66 PMC3136051

[124] Ellis T, Cavanaugh JT, Earhart GM, Ford MP, Foreman KB, Fredman L, et al. (2011). Factors associated with exercise behavior in people with Parkinson disease. Phys Ther, 91:1838-1848. 22003171 10.2522/ptj.20100390 PMC3229047

[125] Tanaka K, Quadros AC, Jr., Santos RF, Stella F, Gobbi LT, Gobbi S (2009). Benefits of physical exercise on executive functions in older people with Parkinson’s disease. Brain Cogn, 69:435-441. 19006643 10.1016/j.bandc.2008.09.008

[126] Schenkman M, Hall DA, Baron AE, Schwartz RS, Mettler P, Kohrt WM (2012). Exercise for people in early- or mid-stage Parkinson disease: a 16-month randomized controlled trial. Physical therapy, 92:1395-1410. 22822237 10.2522/ptj.20110472 PMC3488266

[127] Crizzle AM, Newhouse IJ (2006). Is physical exercise beneficial for persons with Parkinson’s disease? Clin J Sport Med, 16:422-425. 17016120 10.1097/01.jsm.0000244612.55550.7d

[128] States RA, Spierer DK, Salem Y (2011). Long-term group exercise for people with Parkinson’s disease: a feasibility study. J Neurol Phys Ther, 35:122-128. 21934373 10.1097/NPT.0b013e31822a0026

[129] Goodwin VA, Richards SH, Taylor RS, Taylor AH, Campbell JL (2008). The effectiveness of exercise interventions for people with Parkinson’s disease: a systematic review and meta-analysis. Mov Disord, 23:631-640. 18181210 10.1002/mds.21922

[130] Falvo MJ, Schilling BK, Earhart GM (2008). Parkinson’s disease and resistive exercise: rationale, review, and recommendations. Mov Disord, 23:1-11. 17894327 10.1002/mds.21690

[131] Canning CG, Allen NE, Dean CM, Goh L, Fung VS (2012). Home-based treadmill training for individuals with Parkinson’s disease: a randomized controlled pilot trial. Clin Rehabil, 26:817-826. 22257506 10.1177/0269215511432652

[132] Jang Y, Kwon I, Song W, Cosio-Lima LM, Taylor S, Lee Y (2018). Modulation of mitochondrial phenotypes by endurance exercise contributes to neuroprotection against a MPTP-induced animal model of PD. Life Sci, 209:455-465. 30144449 10.1016/j.lfs.2018.08.045

[133] Rezaee Z, Marandi SM, Alaei H, Esfarjani F, Feyzollahzadeh S (2019). Effects of Preventive Treadmill Exercise on the Recovery of Metabolic and Mitochondrial Factors in the 6-Hydroxydopamine Rat Model of Parkinson’s Disease. Neurotox Res, 35:908-917. 30820889 10.1007/s12640-019-0004-x

[134] Kelly NA, Ford MP, Standaert DG, Watts RL, Bickel CS, Moellering DR, et al. (2014). Novel, high-intensity exercise prescription improves muscle mass, mitochondrial function, and physical capacity in individuals with Parkinson’s disease. J Appl Physiol, 116:582-592. 24408997 10.1152/japplphysiol.01277.2013 PMC4073951

[135] Karnia MJ, Myslinska D, Dzik KP, Flis DJ, Ciepielewski ZM, Podlacha M, et al. (2018). The Electrical Stimulation of the Bed Nucleus of the Stria Terminalis Causes Oxidative Stress in Skeletal Muscle of Rats. Oxid Med Cell Longev, 2018:4671213. 29955246 10.1155/2018/4671213 PMC6000852

[136] Marusiak J, Fisher BE, Jaskolska A, Slotwinski K, Budrewicz S, Koszewicz M, et al. (2019). Eight Weeks of Aerobic Interval Training Improves Psychomotor Function in Patients with Parkinson’s Disease-Randomized Controlled Trial. Int J Environ Res Public Health, 16:880. 10.3390/ijerph16050880 PMC6427316 30861998

[137] Silva AZD, Israel VL (2019). Effects of dual-task aquatic exercises on functional mobility, balance and gait of individuals with Parkinson’s disease: A randomized clinical trial with a 3-month follow-up. Complement Ther Med, 42:119-124. 30670228 10.1016/j.ctim.2018.10.023

[138] Zhu M, Zhang Y, Pan J, Fu C, Wang Y (2020). Effect of simplified Tai Chi exercise on relieving symptoms of patients with mild to moderate Parkinson’s disease. J Sports Med Phys Fitness, 60:282-288. 31665879 10.23736/S0022-4707.19.10104-1

[139] Fleisher JE, Sennott BJ, Myrick E, Niemet CJ, Lee M, Whitelock CM, et al. (2020). KICK OUT PD: Feasibility and quality of life in the pilot karate intervention to change kinematic outcomes in Parkinson’s Disease. PloS one, 15:e0237777. 32903267 10.1371/journal.pone.0237777 PMC7480843

[140] Cherup NP, Buskard ANL, Strand KL, Roberson KB, Michiels ER, Kuhn JE, et al. (2019). Power vs strength training to improve muscular strength, power, balance and functional movement in individuals diagnosed with Parkinson’s disease. Exp Gerontol, 128:110740. 31648006 10.1016/j.exger.2019.110740

[141] Vieira de Moraes Filho A, Chaves SN, Martins WR, Tolentino GP, de Cassia Pereira Pinto Homem R, Landim de Farias G, et al. (2020). Progressive Resistance Training Improves Bradykinesia, Motor Symptoms and Functional Performance in Patients with Parkinson’s Disease. Clin Interv Aging, 15:87-95. 32158202 10.2147/CIA.S231359 PMC6986410

[142] Postuma RB, Anang J, Pelletier A, Joseph L, Moscovich M, Grimes D, et al. (2017). Caffeine as symptomatic treatment for Parkinson disease (Cafe-PD): A randomized trial. Neurology, 89:1795-1803. 28954882 10.1212/WNL.0000000000004568 PMC5664303

[143] Simon DK, Wu C, Tilley BC, Wills AM, Aminoff MJ, Bainbridge J, et al. (2015). Caffeine and Progression of Parkinson Disease: A Deleterious Interaction With Creatine. Clin Neuropharmacol, 38:163-169. 26366971 10.1097/WNF.0000000000000102 PMC4573899

[144] Postuma RB, Lang AE, Munhoz RP, Charland K, Pelletier A, Moscovich M, et al. (2012). Caffeine for treatment of Parkinson disease: a randomized controlled trial. Neurology, 79:651-658. 22855866 10.1212/WNL.0b013e318263570d PMC3414662

[145] Fan D, Alamri Y, Liu K, MacAskill M, Harris P, Brimble M, et al. (2018). Supplementation of Blackcurrant Anthocyanins Increased Cyclic Glycine-Proline in the Cerebrospinal Fluid of Parkinson Patients: Potential Treatment to Improve Insulin-Like Growth Factor-1 Function. Nutrients, 10:714. 10.3390/nu10060714 PMC6024688 29865234

[146] Tamtaji OR, Taghizadeh M, Aghadavod E, Mafi A, Dadgostar E, Daneshvar Kakhaki R, et al. (2019). The effects of omega-3 fatty acids and vitamin E co-supplementation on gene expression related to inflammation, insulin and lipid in patients with Parkinson’s disease: A randomized, double-blind, placebo-controlled trial. Clin Neurol Neurosurg, 176:116-121. 30554092 10.1016/j.clineuro.2018.12.006

[147] Taghizadeh M, Tamtaji OR, Dadgostar E, Daneshvar Kakhaki R, Bahmani F, Abolhassani J, et al. (2017). The effects of omega-3 fatty acids and vitamin E co-supplementation on clinical and metabolic status in patients with Parkinson’s disease: A randomized, double-blind, placebo-controlled trial. Neurochem Int, 108:183-189. 28342967 10.1016/j.neuint.2017.03.014

[148] Barichella M, Cereda E, Pinelli G, Iorio L, Caroli D, Masiero I, et al. (2019). Muscle-targeted nutritional support for rehabilitation in patients with parkinsonian syndrome. Neurology, 93:e485-e496. 31278117 10.1212/WNL.0000000000007858

[149] Hiller AL, Murchison CF, Lobb BM, O’Connor S, O’Connor M, Quinn JF (2018). A randomized, controlled pilot study of the effects of vitamin D supplementation on balance in Parkinson’s disease: Does age matter? PloS one, 13:e0203637. 30256811 10.1371/journal.pone.0203637 PMC6157857

[150] Suzuki M, Yoshioka M, Hashimoto M, Murakami M, Noya M, Takahashi D, et al. (2013). Randomized, double-blind, placebo-controlled trial of vitamin D supplementation in Parkinson disease. Am J Clin Nutr, 97:1004-1013. 23485413 10.3945/ajcn.112.051664

[151] DiFrancisco-Donoghue J, Lamberg EM, Rabin E, Elokda A, Fazzini E, Werner WG (2012). Effects of exercise and B vitamins on homocysteine and glutathione in Parkinson’s disease: a randomized trial. Neurodegener Dis, 10:127-134. 22261439 10.1159/000333790

[152] Nascimento CM, Stella F, Garlipp CR, Santos RF, Gobbi S, Gobbi LT (2011). Serum homocysteine and physical exercise in patients with Parkinson’s disease. Psychogeriatrics, 11:105-112. 21707858 10.1111/j.1479-8301.2011.00356.x

[153] Lee SH, Kim MJ, Kim BJ, Kim SR, Chun S, Ryu JS, et al. (2010). Homocysteine-lowering therapy or antioxidant therapy for bone loss in Parkinson’s disease. Mov Disord, 25:332-340. 19938151 10.1002/mds.22866

